# The Combination of Micro Diaphragm Pumps and Flow Sensors for Single Stroke Based Liquid Flow Control

**DOI:** 10.3390/s17040755

**Published:** 2017-04-03

**Authors:** Christoph Jenke, Jaume Pallejà Rubio, Sebastian Kibler, Johannes Häfner, Martin Richter, Christoph Kutter

**Affiliations:** Fraunhofer Research Institution for Microsystems and Solid State Technologies EMFT, 80686 Munich, Germany; j.palleja.rubio@gmail.com (J.P.R.); sebastian.kibler@emft.fraunhofer.de (S.K.); johannes.haefner@emft.fraunhofer.de (J.H.); martin.richter@emft.fraunhofer.de (M.R.); christoph.kutter@emft.fraunhofer.de (C.K.)

**Keywords:** micropump, flow sensor, flow control, single stroke, sensor velocity, differential pressure based flow sensor, thermal flow sensor, damping

## Abstract

With the combination of micropumps and flow sensors, highly accurate and secure closed-loop controlled micro dosing systems for liquids are possible. Implementing a single stroke based control mode with piezoelectrically driven micro diaphragm pumps can provide a solution for dosing of volumes down to nanoliters or variable average flow rates in the range of nL/min to μL/min. However, sensor technologies feature a yet undetermined accuracy for measuring highly pulsatile micropump flow. Two miniaturizable in-line sensor types providing electrical readout—differential pressure based flow sensors and thermal calorimetric flow sensors—are evaluated for their suitability of combining them with mircopumps. Single stroke based calibration of the sensors was carried out with a new method, comparing displacement volumes and sensor flow volumes. Limitations of accuracy and performance for single stroke based flow control are described. Results showed that besides particle robustness of sensors, controlling resistive and capacitive damping are key aspects for setting up reproducible and reliable liquid dosing systems. Depending on the required average flow or defined volume, dosing systems with an accuracy of better than 5% for the differential pressure based sensor and better than 6.5% for the thermal calorimeter were achieved.

## 1. Introduction

The field of micropump research is by now about 30 years old and started with Smits’ patent on a silicon micropump 1984 [1] and the publications of van Lintel in 1987 [2] and Smits in 1989 [3]. Since then, many review papers described different actuation and valve technologies, pump concepts and potential applications mainly in medical devices but also in analysis systems, biological research and other areas [4,5,6,7,8,9,10,11].

However, compared to the potential, not many applications have yet been realized. This may be due to the challenging requirements in performance, efficiency, costs and reliability for most applications [8,10]. Summarizing the applications’ main requirements of performance and reliability, the challenge lies in creating a stable flow or dose a precise amount of volume with micropumps. Many influences such as gas capacitances [12,13,14], particles [12,14,15,16], pressure [17,18], temperature [19], piezo actuator fatigue [20], etc. have to be controlled in order to achieve high dosing accuracy. These obstacles were already evident in an early phase of micropump research, which lead to the combination and integration of microfluidic flow sensors in the relevant range of nL/min to mL/min. Various flow sensor principles have since been established and used for combination with micropumps: thermal anemometers [21,22,23], differential pressure sensing [24,25,26,27], time-of-flight pressure sensing [28], ultrasonic transducers [29], optical [27,30,31] or capacitive [32] flank monitoring, capacitive [33] or impedance based [34] volume monitoring, Coriolis force [35], hotwire [36] and fluid drag force on cantilevers [37,38]. The flow monitoring methods were in part used to investigate micropump behavior and in part connected to a control unit in order to build closed-loop controlled systems to improve dosing accuracy. However, little insight on transient flow sensor accuracy at pulsatile flows was gained. Besides direct flow measurement, other sensors were incorporated into the devices and setups in order to measure secondary influences like pump chamber pressure [36,39,40] and diaphragm deflection [39,41] or environmental influences. Of course, these types of sensors, measuring only influence aspects of possible flow rate changes, have limited validity for flow determination. However, even though the real-time flow is the desired information for a safe closed-loop controlled system, there may be cases where accuracy requirements can be addressed with an environmental or deflection sensor, that is often cheaper due to a higher level of development, less complexity or higher volumes.

Our overall goal is to establish accurate micro dosing systems with high performance and variability of flow to enable applications such as insulin dosing [40] or oil lubrication [32]. For high system accuracy, we want to combine micropumps with flow sensing technologies. However, the pulsatility of micropump flow is a challenge for sensors and dynamic accuracy limited.

Our research goal is to evaluate sensor accuracy at pulsatile flow and to combine sensor and micropump to establish single stroke based flow control. Therefore, we perform calibration measurements with two suitable sensor technologies—differential pressure based flow sensors and thermal calorimetric flow sensors—with a new method, where displacement volumes are compared to flow volumes. We assess sensor robustness and influence of resistive and capacitive damping to combine these sensor technologies with micro diaphragm pumps. For a broad applicability of the control mode, we determine limitations of accuracy and performance for the single stroke based flow control mode.

Within the Introduction, we provide a review of working principle and state of the art of the three main categories for this paper, being the micropump, the sensor technologies and flow control modes. In Section 2, we then explain parameters and fabrication methods of pumps and sensors, and calibration methods. In the Results section, we describe the influence of resistive and capacitive damping, single stroke calibration of sensor technologies and limitation of accuracy and performance for the single stroke based flow control. Sensor accuracy and system performance limitations is discussed in Section 4, together with an outlook on future investigations. The research is summarized with the achieved accuracy and main conclusions.

### 1.1. Micro Diaphragm Pump

**Figure 1:**
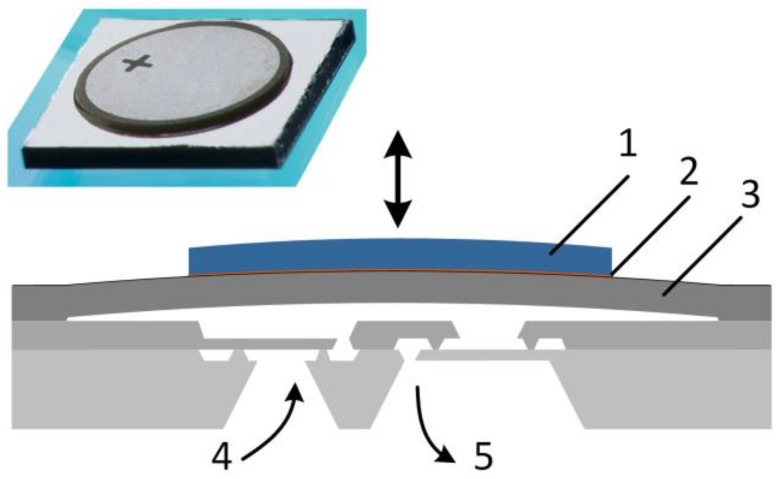
Micropump and its cross-section: (**1**) piezo; (**2**) adhesive; (**3**) actuation diaphragm; (**4**) inlet valve; and (**5**) outlet valve.

**Figure 2:**
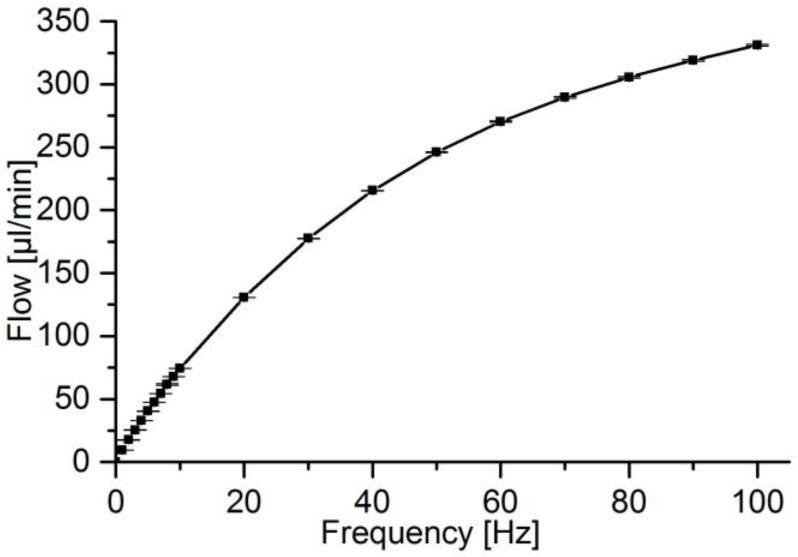
Micropump flow rate over frequency of pump as described in Section 2.1.1.

The type of micropumps addressed in this article feature a micro diaphragm actuator and passive valves. Various actuation principles are plausible, like electrostatic, magnetic or piezoelectric [9]. However, for diaphragms in the range of mm, the piezoelectric actuation delivers the highest forces. For passive valves, the most common are mechanical check valves like flaps and membranes or non-mechanical nozzle-diffuser valves, while the former provide higher flow directing efficiency [9]. The cross-section of the used micropump is shown in Figure 1. The working principle can be separated into the two phases supply and pump mode. In the supply mode, suction pressure is generated by the upmoving diaphragm leading to inlet flow. In the pump mode, the fluid is pushed out through the outlet valve by excess pressure generated by the downward moving diaphragm. This behavior causes the pulsatile flow behavior at the pump outlet. A rectangular driving signal allows for the highest fluidic performance with increasing frequency, displayed in Figure 2, because the fluid is moved in the smallest possible time.

### 1.2. Miniaturizable Inline Flow Sensor Technologies

In order to create accurate closed-loop controlled micro dosing systems, the sensors have to fulfill a number of requirements. The sensors need to be placeable in-line of the fluidic path and provide electronically readable real-time flow information. The feasible miniaturized size should be comparable to the micropump to maintain the size advantage on a system level. Most important, the sensor needs to be able to measure the highly pulsatile transient micropump flow correctly. The pulsatility of micro diaphragm pump flow leads to high changing velocities of flow rate in a wide range of flow and is a challenge for sensors. A high sensor velocity, being the time between a flow step and the correct sensor signal, is necessary and is summarized for different microflow sensing technologies in Table 1. Meeting the other basic requirements of dimensions, in-line placement and electronic readout, the differential pressure based (DPB) and the thermal flow sensing principles were selected and their state of the art to accurately measure the pulsatile flow reviewed.

#### 1.2.1. Differential Pressure Based (DPB) Flow Sensor

This type of flow sensor uses a differential pressure between two points in the fluidic path while knowing the flow resistance to calculate the flow. In general, a flow resistance limits the fluidic performance of the system and should therefore be minimized. However, reducing the flow resistance and therefore the pressure drop also reduces the exploitable signal for the sensor. Using very sensitive pressure sensors can compensate that theoretically up to a lower limit, but makes the system vulnerable to flow misinterpretation due to pressure variations from external influences. An equilibrium, where externally induced noise and sensor zero stability are in the same order of magnitude should be chosen. Pressure sensors in Wheatstone-bridge configurations provide minimized dependence on temperature.

**Figure 3:**
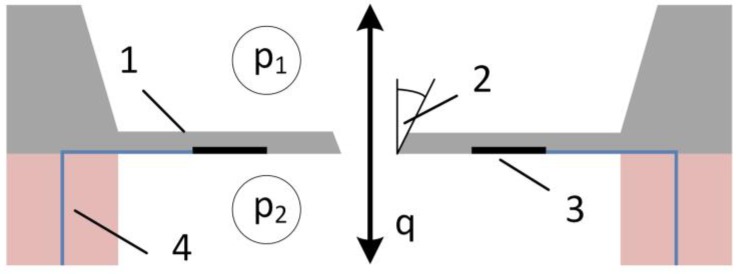
Principle of pressure sensor based flow sensor: (**1**) silicon diaphragm; (**2**) orifice cone angle; (**3**) piezoresistors; and (**4**) conducting gasket.

The most important characteristic for pulsatile flow measurement is the sensor velocity. For flow sensors with piezoresistive solid state pressure sensors, the main constraint for flow sensor velocity is the mechanical response time. As shown in Figure 3, using a diaphragm based differential pressure sensor with a central orifice as flow restriction simplifies the fabrication of such a sensor significantly, and allows for an almost temperature independent flow sensor at large orifices compared to diaphragm thickness. This was patented in 1997 [47] and described by Richter et al. [27]. The authors described the basic principle of the sensor, the influence of orifice diameter, temperature and viscosity on the static behavior, the blocking effect of gas bubbles and some basic considerations on the dynamic behavior with response time and signal validity if used with micropumps.

Depending on the ratio α between orifice diameter and diaphragm thickness, the influence of viscous friction and therefore viscosity and temperature influence changes. The transition from dominating viscous friction at a ratio of α << 1 (Hagen–Poiseuille) to dominating inertia effects with α >> 1 (Torricelli) and a state in between with α = 1 (Hagenbach correction), where diameter and thickness are in the same order of magnitude, can be described by different laws mentioned in brackets [27]. While Hagen–Poiseuille and Torricelli match the static calibration curves well for respected geometries, the intermediate state proves to be less accurate and a certain temperature hence viscosity dependency was observed [27]. Unfortunately, the geometries needed for the investigation of dynamic micropump behavior together with externally induced noise levels feature ratios of a little over one and cannot be fitted very well with the laws.

A FEM study of transient flow through the sensor in the state of the art showed, that velocity profiles behave quasi-static up to harmonic frequencies of 10 kHz [27]. Furthermore an experimental study was carried out, where a steady flow was modulated with a harmonic flow profile in order to investigate the accuracy of accumulated sensor flow compared to balance flow for frequencies up to 1 kHz [27]. This study showed increasing deviations for rising frequencies, hence rising flow rate alteration speed, up to roughly 11% underestimation of flow at 1 kHz. Due to the setup of an elevated modulated flow, the noisy regimes of the sensor close to zero flow were avoided. The investigations on the dynamic behavior also show contradictory results for a 100 Hz driving frequency. While average flow of sensor signal and reference balance show an underestimation of flow, comparing it to an FEA-simulations showed almost no phase shift but an overestimation around the peak flow.

#### 1.2.2. Thermal Flow Sensors

**Figure 4:**
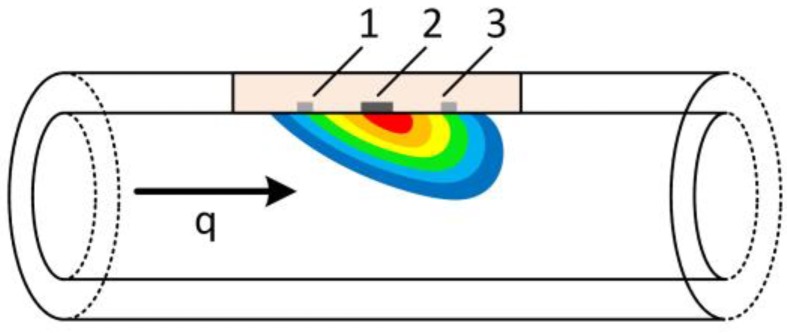
Principle of thermal flow sensor: (**1**,**3**) temperature sensor; and (**2**) heater.

Thermal flow meters, where local heating of the fluid is used, are the most common sensors to monitor microflow. They are based on three different configurations, which are heat loss at hotwires, microthermotransfer based calorimeter and thermal TOF (time-of-flight) [44,48]. While some measure the mass flow and others measure the flow velocity, all are sensitive to thermal properties of the fluid. Thermal transduction principles are thermoresistive, thermoelectric, thermoelectronic or frequency analog [48]. While calorimeter, as depicted in Figure 4, are composed of one heater and at least two temperature sensors (one upstream and one downstream), they are driven in a steady state of heater power. Based on the temperature difference and the heater power consumption the current flow rate can be determined [49]. For thermal TOF sensors, the time difference between heat pulse generation and temperature peak detection at the sensor is a measure of flow velocity. While calorimeter are better suited for flows with faster thermal diffusion than convection, the thermal TOF principle brings advantages at higher flow velocities [21]. The thermal conduction between heater and thermosensors should be minimized by reducing thermal capacity and using isolating materials. Using freely suspended nitride channels [50] or removing bulk substrate behind the sensing elements [44] are measures to achieve this. For the measurement of pulsatile flow, the calorimetric principle is more suitable due to a constant and higher readout-frequency compared to the exponentially increasing flight times and decreasing accuracy for declining flows rates at TOF sensors. Therefore, low flow pulses can be monitored more accurately with calorimeters. In order to increase sensor velocity the distance between heater and sensor could be reduced. However, this stands in contrast to the sensitivity [49] as the solid-state heat transfer disturbs the temperature sensor, limiting both calorimeters and TOF sensors. Except for one combination of thermal TOF and a micropump [51], mainly calorimetric flow sensors were used for combination with micropumps [22,23,51,52]. Woitschach et al. mentioned significantly rising deviations for increasing flow rates due to the pulsatility of the micropump without going into detail explaining those [23]. Except for the latter, no investigations about the dynamic accuracy of calorimetric flow sensors are known to the authors.

### 1.3. Flow Control Methods

**Figure 5:**
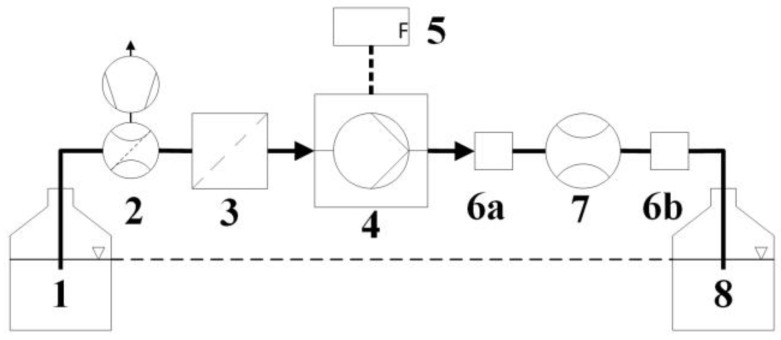
Generic liquid dosing system setup for stable continuous flow: (**1**) reservoir; (**2**) inline degasser; (**3**) filter; (**4**) micropump; (**5**) electronic driver; (**6a**,**b**) pressure smoothing element (PSE); (**7**) flow sensor; and (**8**) outlet reservoir.

To start with, the system setup sets the foundation for a reliable and accurate dosing system. In Figure 5, a generic setup for microfluidic dosing systems is shown. Besides the reservoirs, the system can consist of a degasser, filter, pump with driving unit, PSEs (pressure smoothing elements) and a flow sensor. The degasser has two purposes, first to remove passing bubbles and second to degas the liquid so that remaining bubbles in the fluidic path are dissolved after several minutes of operation. An alternative method to provide an initially bubble free fluidic path is deploying the CO_2_-priming method as described in [53]. Little variations in inner cross section diameter supports this strategy. The filter prevents particles from entering sensitive structures. In the pump, particles can lead to valve leakage or stroke obstruction, for sensors, clogging is the most critical issue. The micropump needs driving electronics to provide adjustable voltage levels and signal shapes with frequencies up to several kHz. Depending on the desired control mode, different pressure smoothing elements need to be implemented. The flow sensor provides the basis for accurate flow control and can be used for additional safety as bubble detector. The real output flowrate can only be measured behind the pump, as leakage may occur at the interconnections of the pump to the fluidic path. Therefore, the preferred location is after the pump and close to the outlet. For investigation purposes, it is recommended to place two sensors, one before and one behind the pump, to compare both signals. For maximum performance, as little flow resistance as possible is to be installed, mainly concerning filter and flow sensor. A free flow protection valve and a flow sensor for leakage detection with lower flow range offer additional safety.

## 2. Materials and Methods

One of the main advantages of micro diaphragm pumps is the high range of flow rate that can be adjusted by changing the driving signal. To ensure a high accuracy while combining micropump and flow sensor, three different modes of operation are possible. The first provides a steady flow for the sensor, where static calibration curves are valid. To achieve such a stable flow with a micropump, a carefully designed damping needs to be implemented. The range of driving frequencies to stay within small boundaries of flow variation, however are quite small. By exceeding these frequency boundaries, the pulsatility of the flow will increase, leading to the second mode. Transient dynamic accuracy of DPB and calorimetric sensors however is also limited, mainly undetermined and no model for a transient correction is known. This leads to the third imaginable operating mode, where fully completed single strokes of pulsatile flow are measured. This mode offers certain advantages. The minimal volumes of each stroke that can be dosed can be adjusted by the voltage levels and the average flow can be adjusted in a wider range until reaching the cut-off frequency. Furthermore, the accuracy for these single strokes can be measured and calibrated for a pump-sensor combination. For a wide frequency range, the capacitive damping has to be minimized. Therefore, between pump and sensor, gas capacitive damping needs to be prevented, as shown in Section 3.1 and elastic capacitive damping needs to be minimized, as described in Section 3.2. 

### 2.1. Materials and Equipment

Table 2 shows an overview of the used materials and equipment. It also states whether we fabricated it or if it is commercially available. Design parameters and fabrication processes for micropumps and DPB flow sensors are described subsequently.

#### 2.1.1. Silicon Micropump

The key parameters of the silicon micropump, with its cross-section sketched in Figure 1, are shown in Table 3. The process to fabricate the micropump is as follows: the pump consists of three wafers, two for the valve and one for the actuator including the pump chamber, as indicated in Figure 1. The monocrystalline silicon wafers start with mask layers of silicon nitride and silicon oxide. After lithography and DRIE etch masking, the main structuring is done by KOH etching. A two-step etching process allows for different structuring depths of the valve wafers. After removing remaining mask layers, thermal oxidation allows for the silicon fusion bond in order to join first the two valve wafers and subsequently the actuation wafer. To reduce the diaphragm thickness, grinding and polishing is used. A sputtered aluminum layer provides the contact for the adhered piezo disc from manufacturer PI. The piezo is attached to the diaphragm according to a fabrication method that is also referred to as initial deflection method [56] to achieve a higher compression ratio by applying an initial deflection combined with low pump chamber height. The pump is adhered to a fluidic adapter that connects to the flow sensors by use of stiff PEEK capillaries.

#### 2.1.2. Differential Pressure Based (DPB) Flow Sensor

For the manufacturing of the DPB flow sensor, a bi-directional 5 psi differential pressure sensor for liquids was chosen, providing a response time of 1 ms. The chosen sensor type was of the Honeywell 24 PC Series, while the Sensortechnics/FirstSensor RPOP005D6A presents a slightly less accurate alternative. A central orifice was lasered into the sensor diaphragm, using a 355 nm UV laser in pulsed mode at 20 kHz and 2 W for 1 s and a circular oscillation pattern. Diameters and respective maximum flow rates at the maximum pressure of the sensors are displayed in Table 4. The cone angle, originating in the lasering process, results in a direction dependent flow number μ. The nozzle diameter is always the larger one, leading to a nozzle like flow profile if pressure is applied at this side of the diaphragm. That anisotropy can either be avoided by use of DRIE etching or enhanced and used to measure leakage flow with higher sensitivity.

Pressure sensors are naturally very sensitive to internal or externally induced stress, leading to measurably changing zero-flow offset values. That is why, besides calibration measurements, an additional zero-offset recording has to take place once the sensor is installed in the final setup. For flow calculation, this new zero-offset value has to be the reference value for signal values as if it was the zero-offset value of the calibration measurement. The offset values were individually evaluated before each measurement and exhibit a standard deviation of 3.6 μL/min.

### 2.2. Measurement Methods

**Figure 6:**
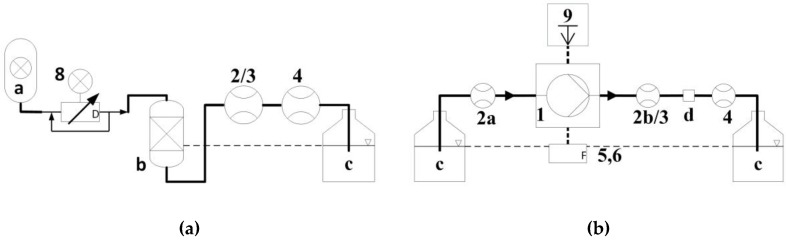
Measurement setups for: (**a**) static calibration; and (**b**) single stroke calibration (Table 5). Description numbers refer to Table 2. Additionally: a: pressure source; b: pressured reservoir; c: reservoir; and d: PSE (pressure smoothing element).

Table 5 shows the overview over the methods used for measurements and data processing. For Methods (a) and (b), the setup structure is sketched in Figure 6. The medium for all measurements was DI water. The carbon dioxide priming method [53] is used to ensure a bubble free fluidic path between reservoir and outlet and was applied for all measurements.

#### 2.2.1. Static Calibration of Flow Sensors

The static calibration of the flow sensors was done with the measurement setup shown in Figure 6a. The water reservoir (b) is set under air pressure, controlled by a pressure regulator (a). The readout of the flow sensors (2/3) are compared to a reference flow sensor (4) placed downstream. The pressure was increased until the maximum sensor range had been reached. Three full cycles of pressure increase and decrease were conducted in each direction. The OriginPro (v9.1G, OriginLab Corporation, Northampton, MA, USA) curve “ExpDec2” was fitted to the experimental results and used to calculate the dynamic flow.

#### 2.2.2. Single Stroke Calibration Method

The measurement setup in Figure 6b for the dynamic calibration method consists of a micropump and at least two flow sensors. For the DPB sensor, one was located right before the pump (2a) and one behind (2b). For the thermal calorimeter, only one was placed behind the pump (3). The elastic capacitive damping was minimized by using stiff capillaries and short distances between pump and sensor. The average flow was monitored by the reference flow meter (4). The actuator deflection was measured at the diaphragm center by a profilometer (9) with 4 kHz scanning frequency.

The procedure to acquire one calibration point includes a fully finished pump cycle at a pump driving frequency of 0.5 Hz, providing 1 s each for supply and pump mode. The voltage levels were varied between 200 V and −60 V in 20 V-steps, depending on the sensor range.

**Figure 7:**
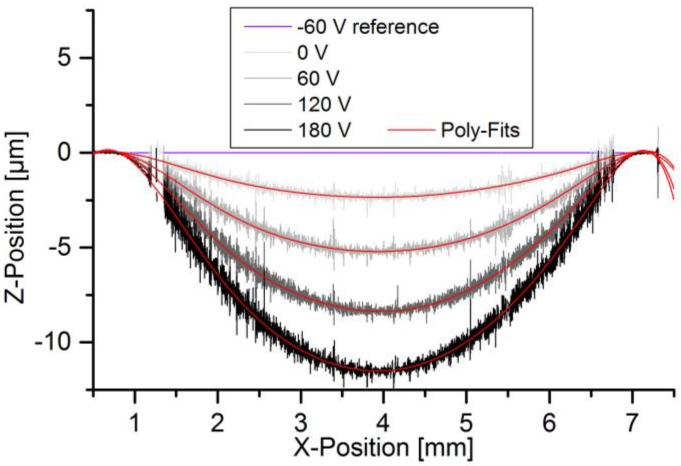
Line-displacement of actuator for stroke volume determination.

The calibration compares two volumes, one is the integrated flow volume of the flow sensor, the other is the calculated stroke volume of the actuator deflection. To calculate the stroke volume of each cycle, the central point deflection is multiplied by a factor representing the volume per deflection:

(1) $$V_{disp}=d_{central}\cdot \frac{\pi \int_{a}^{b}x\cdot y\left(x\right)dx}{d_{line-center}}$$

the volume per deflection is constant within the full deflection range of the micropump. The measured relative line displacement of the actuator, as shown in Figure 7, is used to calculate this ratio by integrating the line fit over half a revolution after shifting the line center to x = 0.

## 3. Results

The achievable system accuracy depends on the sensor accuracy, the time between measuring deviations and adjusting the flow and the adjustment accuracy. The task of dosing systems lies either in generating a certain average flow or dosing a defined volume. Diaphragm micropumps always generate pulsatile flow. The systems outlet flow characteristic can be fully pulsatile, meaning the flow always reaches zero each cycle, continuous, by implementing tailored PSE (pressure smoothing elements), or in a state in between. To work with the single stroke based flow control, the sensor is placed before the optional main PSE. The influence of capacitive and resistive damping on flow pulsatility is evaluated in Section 3.1. The ability of DPB and thermal flow sensors to measure the transient single stroke micropump flow is investigated in Section 3.2, followed by describing the influence of these results on single stroke based closed-loop control.

### 3.1. Capacitive and Resistive Fluidic Damping

Resistive fluidic damping depends on the flow resistance between actuator and the whole fluidic path. Resistive damping reduces flow velocity and prolongs the flow, which reduces the maximum driving frequency, where flow pulses are fully finished.

Capacitive fluidic damping in general has the same smoothing effect on a flow pulse as resistive damping, but stores the pressure in a capacitance. Such a capacitance can be a bubble within the fluidic path or an elastic element. Decoupling the actuator from a high fluidic resistance or fluid inertia by using a capacitance, increases the maximum driving frequency at fully finished flow pulses but reduces the stroke volume due to the counter pressure at the charged capacitance. During the time of the supply mode, the capacitance pressure is transformed to outlet flow. In a correct configuration, this can increase maximum flow, while smoothing it.

However, it is very difficult to implement a reproducible capacitive damping if different sized bubbles may occur in the fluidic path, which is why gas bubbles need to be avoided. Capacitive damping in general can never completely be avoided, as even stiff solid-state bodies such as capillaries or other housing parts exhibit a certain elasticity. Elastic capacitive damping though can be better controlled by specific design.

**Figure 8:**
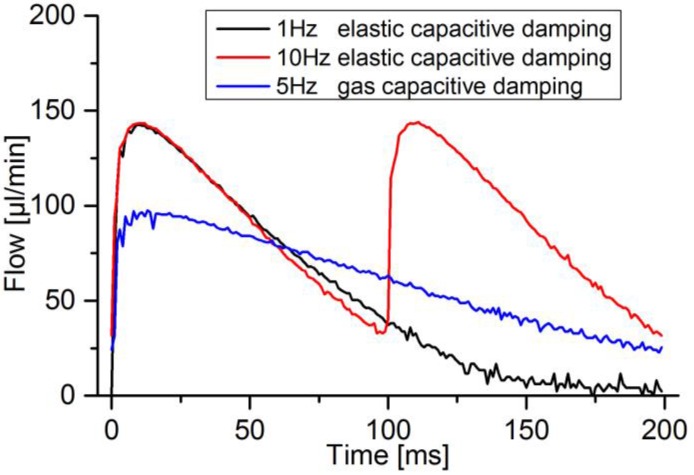
Influence of elastic and gas capacitive damping on the flow.

To investigate the magnitude of gas capacitive and elastic capacitive damping on the flow, a perfectly filled fluidic path with as little elastic damping as possible was set up together with a DPB flow sensor. The elastic damping between pump and sensor lead to finished flow pulses after 200 ms, driven at 1 Hz (Figure 8). At 10 Hz, leaving 50 ms for the pump mode and 100 ms for the whole cycle, the pulse was not able to finish completely. After introducing a bubble in between pump and sensor, the flow was measured at 5 Hz meaning the pump stroke is limited to 100 ms, the full cycle to 200 ms. At pure elastic damping, the charged capacitance only results in minor deviations of flow curves, while strong smoothing occurs at gas capacitive damping

PSEs (pressure smoothing elements) allow for tailored capacitive elastic damping. Options for implementation are elastic solid-state elements like diaphragms, chambers or tubes. Here, PSEs can be designed easily with available models of basic geometries and materials. Materials should exhibit low permeability for gases as longer operation downtimes of several hours or days may lead to evaporation through thin plastic structures or diffusive materials like silicone, leaving bubbles in the fluidic path behind.

### 3.2. Transient Single Stroke Measurement

To measure the transient flow of the fluidic step response of a rectangular driving signal accurately and in full range is a challenge for sensors due to high flow rate changing rates. The introduced sensor technologies—differential pressure based flow sensors and thermal calorimeter—are the fastest in-line measurement methods while providing electrical readout that were identified with sensor velocities of 1 ms for the DPB sensor and 40 ms for the thermal sensor.

**Figure 9:**
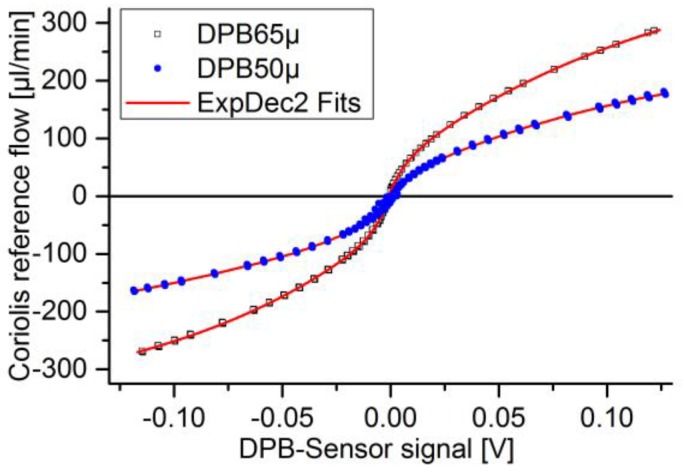
Bi-directional calibration curves including mathematical fit-functions.

**Figure 10:**
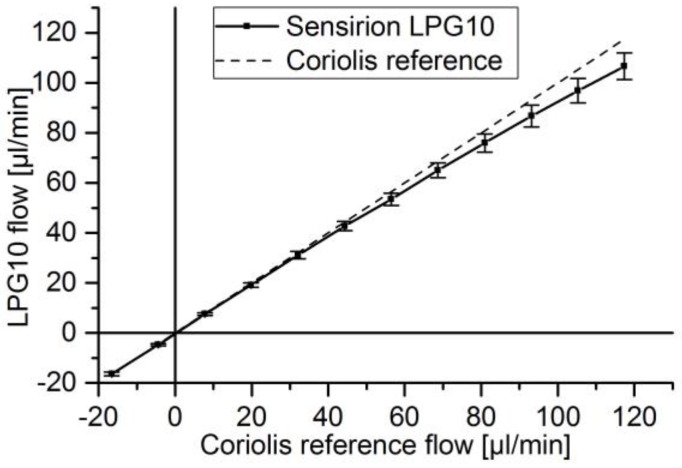
Static calibration of thermal calorimeter (LPG10) against coriolis flow meter.

Static calibration measurements with the same reference flow sensor were taken out to establish comparable flow values Section 2.2.1 and to describe the achievable accuracy in static operation. For the DPB sensor a bidirectional static calibration curve was recorded (Figure 9). The high steepness of the curve close to zero indicates low sensitivity. This leads to a high inaccuracy for low flow rates, but increasing accuracy for rising flow. The direct static calibration curve with sensor signal over flow cannot be determined, as we have to rely on a program output. However, from the literature, it is known that an exponential curve is to be expected for thermal calorimeter [44]. This correspond to the increasing deviations with rising flow rates as observable in Figure 10. The sensor behaves bidirectional, which is only indicated for a short range in the diagram.

**Figure 11:**
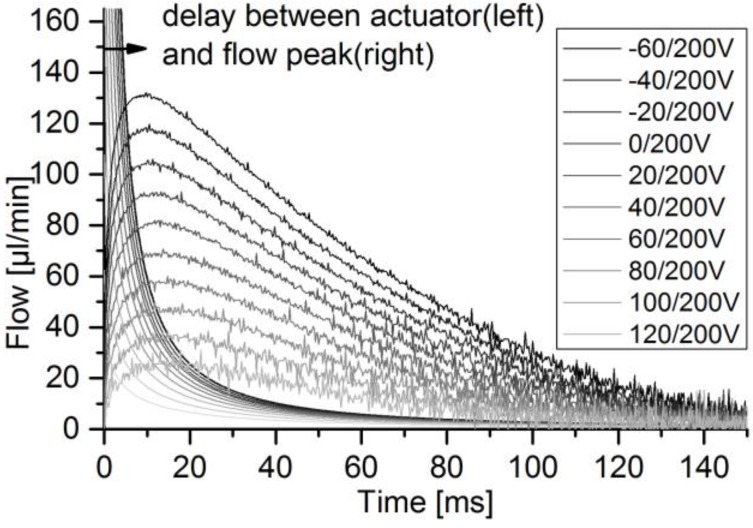
The actuator velocity (derivate of displacement Hill-fit-curves of Figure 13) and the DPB50µ sensor flow compared for the pump mode.

**Figure 12:**
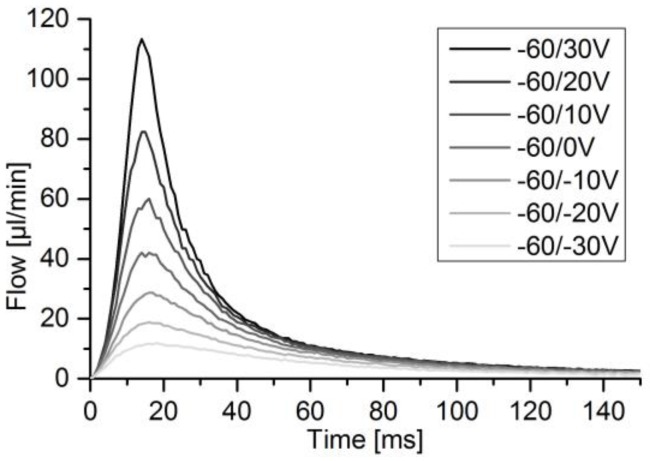
Dynamic flow pulse for rising voltage levels.

For the determination of sensor accuracy of pulsatile flow, the micropump was used to generate flow pulses of different height and volume by increasing the absolute voltage difference, according to the method in Section 2.2.2. The DPB sensor was addressed with the pump’s full voltage amplitude of 260 V. The thermal sensor was addressed with a maximum of 90 V. The actuator velocity peak is always reached within 1 ms. Comparing the sensor signal to the actuator velocity, as depicted in Figure 11, a delay of 8–14 ms from actuator to flow signal peak occurs, depending on the voltage level and for a perfectly filled fluidic path. The thermal sensor reaches its flow peaks at relatively stable 20 ms (Figure 12). DPB flow finishes after 150–200 ms, while calorimetric flow finishes after 100–150 ms.

**Figure 13:**
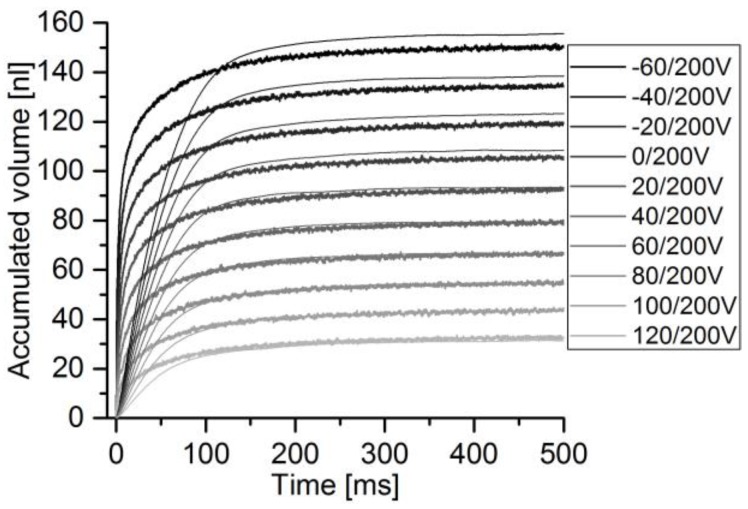
Comparison of accumulated stroke volume (noisier signal) and DPB50µ sensor flow.

**Figure 14:**
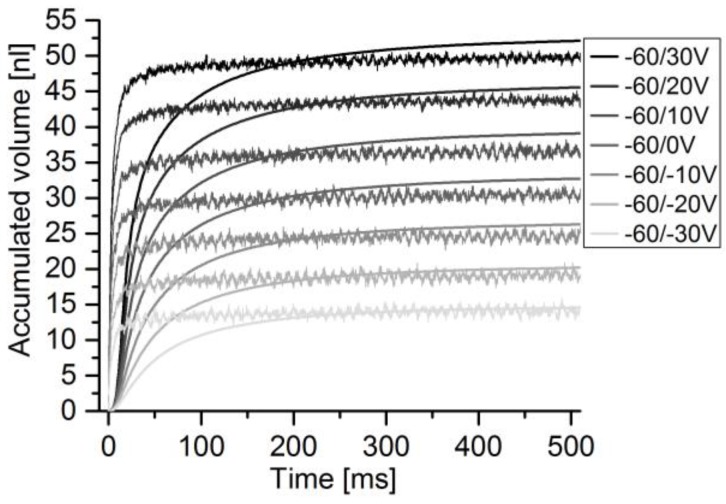
Comparison of accumulated stroke volume (noisier signal) and LPG10 sensor flow.

For the determination of average flow and flow volumes, the accumulated flow is calculated for each pump stroke. The time course of the stroke volume and the integrated sensor flow are compared and depicted in Figure 13 for the DPB50μ sensor and in Figure 14 for the thermal sensor. While the actuator signal can be calculated for the whole measured spectrum, the DPB sensor’s flow signal becomes too noisy to be evaluated for pulses with peak flows below 25 μL/min for sensors with maximum flow rates of −165 μL/min. With full-scale flow of 120 μL/min of the thermal sensor combined with high accuracy at low flow rates, lower pulses down to can be measured.

**Figure 15:**
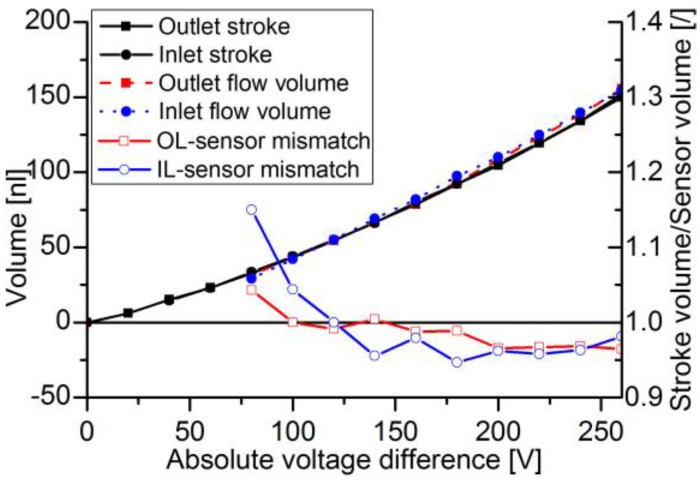
Accumulated volume for stroke (y-error: 0.09–0.19 nL) and flow (y-error: 1.79–2.26 nL) at 500 ms and the mismatch factor between both.

**Figure 16:**
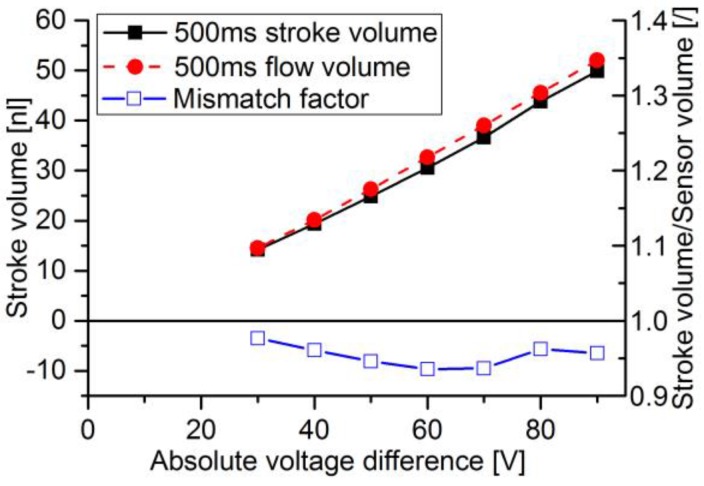
Comparison of stroke volume and sensor volume (y-error: 0.32–0.57 nL) at 500 ms for the thermal flow sensor.

Stroke volume and sensor flow volume are compared at 500 ms, when the actuator is static and all pressure capacitances are equalized, hence no more liquid is flowing. For the two DPB sensors, deviations between the two volumes are shown in Figure 15. For voltage differences below 80 V, the signal was too noisy to evaluate. The sensors underestimate the flow up to voltage differences of 120 V, after that an overestimation of flow stabilizes at roughly 4–5%. The thermal flow sensor was subject to much higher flow rate changing rates. Stroke and flow volume are compared in Figure 16 and show a general overestimation of flow between 2–6.5%.

### 3.3. Single Stroke Based Flow Control Mode

Applying the single stroke based control mode has certain limitations for performance and accuracy. The volume of one pump stroke is mainly limited by the maximum electric field that can be applied to the piezo and is typically between −0.4 kV/mm and +2 kV/mm. That is −60 V/+300 V for our piezo thickness of 150 μm. However, the actuator touches the pump chamber boundary at 200 V, which gives the practical limit. Before defining the maximum frequency, the main limiting factors on a minimal setup are described briefly. The outlet path starts with the actuation diaphragm and the outlet valve within the pump followed by a minimized elastic capacitive damping element, an in-line flow sensor and an outlet tube. The whole outlet path exhibits a certain fluidic resistance, significantly increased by the DPB sensor compared to the thermal sensor. The actuation displacement flow is mainly determined by the resistance and the charged pressure at the elastic capacitance between pump and sensor. The flow through the sensor is further damped compared to the displacement flow due to the capacitance but effectively finishes at the same time as the actuator. The final sensor readout is additionally smoothed and delayed by the sensor velocity. To define the maximum driving frequency of the pump to stay within fully finished flow pulses, all these time delays have to be considered. If one wants the actuator to balance itself out to fully static, the maximum frequency is:

(2) $$f_{\max static}={\left[2\left(t_{disp\;static}+t_{sensor\;velocity}\right)\right]}^{-1}$$

For maximum performance at a certain elastic capacitive damping, only the measured flow needs to return to zero within a full cycle. That means the actuator is switched from pump to supply mode, while still moving downwards. In this case, the maximum frequency for single stroke based control is limited to:

(3) $$f_{\max dyn}={\left[\left(t_{disp\;dyn}+t_{capacitive\;delay}+t_{sensor\;velocity}+t_{processing}\right)\right]}^{-1}$$

While the processing time can be done in the supply mode for the static scenario, it has to be added to Equation (2) for real-time flow control. A stiff pressure-insensitive actuator increases the performance by achieving higher stroke volume at backpressure. If a continuous flow is desired, a PSE can be placed after the sensor. The benefit lies in establishing a continuous flow while maintaining the pump cycle based closed-loop control. This also decreases the maximum flow range needed for the sensor, which therefore increases sensitivity, as the ratio between both is limited.

The limitations of achievable accuracy depend on the adjustment accuracy and the sensor accuracy. The sensor can measure each stroke with a certain accuracy (acc) and precision (prec). The sensor’s accuracy and precision is stroke volume dependent. With rising stroke numbers N_strokes_ for the dosing task, the precision influence gets smaller. The total accuracy of the stroke adjustment only plays a role for the last stroke, as it can be set according to the accumulated flow volume before. The total accuracy for each dosing task is therefore:

(4) $$total\;dosing\;accuracy\;\left[nl\right]=\pm \sum^{\text{​}}\left[acc_{sensor}\left(\Delta U\right)\right]\pm \frac{prec_{sensor}\left(\Delta U\right)}{N_{strokes}}\pm total\;acc_{adjustment}$$

## 4. Discussion

### 4.1. Sensor Accuracy at Pulsed Flow

The goal was to evaluate sensor accuracy of micropump generated pulsatile flow. In general, these pulses exhibit a steep flow increase within a few milliseconds (Figure 11 and Figure 12), followed by a flow decline within tens or hundreds of milliseconds. With increasing absolute voltage difference, the flow pulses show a higher peak flow but similar overall times to reach zero flow. While the investigated sensor technologies are subject to misinterpretation of fast changing flow rates, that effect is diminished, as there is always a rising and a falling flank.

The DPB sensors uniformly showed higher inaccuracy with decreasing flow pulse heights (Figure 15). Below 80 V, the signal was not clearly evaluable, as it was within signal noise. One difficulty of this technology is the choice of pressure range and flow restriction. While a low flow restriction (high orifice diameter) leads to inertia dominated flow with less temperature dependence, the sensor range has to be small enough to measure the full range of the maximum desired flow pulse. However, this leads to a high sensitivity and high signal noise due to environmental influences. Due to the steep static calibration curve close to the offset value, little changes in voltage mean a high difference in flow. Combined with the sensor noise very low flow rates might not be detected but results in high errors. For flow pulses generated with more than 120 V, a stabilizing deviation of roughly 5% is observable. Not all sources of errors are known yet. One already mentioned is an offset shift between calibration and individual measurement. Sources of misinterpretation might be in fast changing flows, where pressure difference does not match the static calibration of actual flow and temperature difference between calibration and measurement. Another error case is the partial clogging of the sensor orifice, which is not easily detectable, because it just compresses the calibration curve, overestimating the actual flow. Due to the fast response time of below 1 ms, the sensor velocity plays a subordinate role in smoothing the signal. This is a major advantage of this sensor type and makes it suitable to investigate highly pulsatile micropump flow.

For the thermal calorimeter, the achieved accuracy of integrated flow over a full pulse was within 6.5%. The static calibration of the thermal calorimeter shows the highest flow precision at zero flow and decreases with rising flow due to an exponential shaped flow over sensor signal (Figure 10). Therefore, lower pulses with respect to maximum flow can be measured compared to the DPB sensor. However, the sensor speed of 40 ms [43] is lower than the 1 ms of the DPB flow sensor, which leads to reaching the flow peak at around 20 ms compared to 8–14 ms for similar elastic damping and despite lower resistance (Figure 12). Heat transfer from liquid to the temperature sensors combined with surrounding thermal mass not only results in the slower sensor velocity, but also reduces the measured peak flow. For rising flow an underestimation and for decreasing flows an overestimation of flow is to be expected. The overall flow overestimation indicated a higher impact of the slower changing decreasing pulse flank. The microthermotransfer principle also allows for higher tube diameters and provides a more robust system with less chance of clogging through bubbles or particles. For a wider flow range combined with high sensitivity for leakage flow detection a specific design or additional sensors can be considered.

### 4.2. Flow Control Modes

To employ the single stroke based flow control on a system level, not only sensor accuracy is critical, but also fluidic performance, efficiency and stroke adjustment accuracy. While the DPB sensor includes a significant additional fluidic resistance in the outlet path with its orifice, the thermal sensor allows for larger tube diameters. The time for finishing the flow pulse across the sensor will decrease for the thermal sensor, the maximum driving frequency to stay in the finished single stroke mode will increase compared to the DPB sensor. As described in Section 3.3, for higher frequencies it is recommended to just have finished flow pulses at the sensor after a full cycle, not at the actuator. However, having unfinished movement of the actuation diaphragm will result in a higher temperature dependence, hence lower stroke volume adjustment accuracy. Additionally the efficiency decreases, because the actuator switches to supply mode before the full deflection is finished. To sum up, higher fluidic performance comes along with lower adjustment accuracy and lower efficiency.

By smoothing the pulsatile micropump flow with large capacitances (PSEs), holding multiple stroke volumes with low pressure changes, a continuous flow rate can achieved. Sensors with lower velocity can be employed behind the smoothing elements to measure continuous flow. A range of flow rate with a high accuracy can be chosen, being low flow rates for thermal calorimeters or high flows for DPB based sensors. Another advantage over transient pulsatile measurements is the potential direct use of the output parameter for control purposes requiring no or less further processing. However, implementing PSEs for minimal damping for a certain flow rate is challenging. Changing the frequency will result in more pulsatility and less accuracy. At the single stroke based control mode, a higher range of driving frequency can be employed with defined accuracy limits. Dosing defined volumes with several strokes can be done with a very high accuracy by adjusting the stroke volume after each cycle, leaving only the sensor accuracy and the last adjusted stroke as main sources for deviations (compare to Equation (4)). In general, capacitive damping has a big impact on flow, which is why it has to be limited to elastic capacitive damping by avoiding gas bubbles.

### 4.3. Future Investigations

While capacitive damping plays a strong role in flow performance for microfluidic systems, there is little existing literature about designing elastic capacitances of defined magnitude of influence. Mechanical models for different shapes like tubes or diaphragms should be adapted to provide information about their dynamic capacitive behavior with fluid pressure. Thereby, continuous flow control with the sensor placed behind the smoothing element might achieve higher accuracy or even a wider range of frequency with a variable PSE. Single stroke based control mode on the other hand would profit from enabling continuous flow in minimal space requirement. A dynamic sensor calibration method that is able to correct changing flow rates would of course be most desirable and enable an accurate flow control over the full frequency range of micropumps.

## 5. Conclusions

Micro dosing systems based on micropumps are able to accurately deliver defined volumes of liquid down to nanoliters or average flow rates in the range of nL/min to μL/min. Inherent system or micropump fatigue together with variable changes of environmental parameters, however, influence performance stability and reproducibility. Combining micro diaphragm pumps with flow sensors enables accurate closed-loop controlled systems.

In this paper, we evaluated two sensor technologies for their ability to measure the highly pulsatile micropump flow to be employed for single stroke based flow control: differential pressure based (DPB) sensors and thermal calorimetric flow sensors. Depending on the required average flow or defined volume, dosing systems with an accuracy of better than 5% for the differential pressure based sensor and better than 6.5% for the thermal calorimeter were achieved. A method was developed that enables calibration of single stroke flow pulses by comparing displacement volume with sensor volume. Limitations of accuracy and performance at combining these sensors with micropumps for the single stroke based flow control were given. DPB flow sensors exhibit a sensor velocity of 1 ms that results in peak-flow detection after 8–14 ms. However, orifices of 50–65 μm produce significant fluidic resistance, which prolongs the flow and reduced maximum frequencies for single stroke based flow control to 5–6 Hz. Furthermore, a trade-off between particle robustness and temperature dependence versus signal to noise ratio regarding their orifice diameter has to be taken into account. By contrast, thermal flow sensors display higher fluidic performance up to 6–10 Hz maximum frequency and higher particle robustness due to a larger tube diameter and good sensing range combined with high sensitivity. Because of the sensor velocity of 40 ms, the flow peak is only reached after 20 ms.

Enhancing sensor velocity for thermal calorimeter or finding transient calibration methods for both technologies are desirable goals to enable stable, accurate operation up to higher pump frequencies. Alternatively, providing design guidelines of implementing pressure smoothing elements for smoothed flow also enables higher flows with accurate measurement, but provide little flow variability.

## Figures and Tables

**Table 1:** Sensor principle and their measurement velocity.

Sensor Principle	Measurement Velocity
Optical/capacitive flank monitoring	0.4 ms [42]
Differential pressure based	<1 ms [27]
Thermal calorimeter	40 ms [43]
Thermal TOF (time-of-flight)	6–25 ms [44]
Coriolis	50–200 ms [45]
Gravimetric balance	<2 s [46]

**Table 2:** Material and equipment overview.

Materials and Equipment	Manufacturer and Model
Silicon micropumps	Fraunhofer EMFT [54,55]
Differential pressure based (DPB) flow sensor	Fraunhofer EMFT [27]
Thermal flow sensor	Sensirion LPG10-0500
Coriolis mass flow meter	Bronkhorst miniCORI-Flow
Gravimetric balance	Sartorius 225S
Frequency generator	Agilent 33120A
High voltage amplifier	Piezomechanik SVR 150/3
Pressure controller	Mensor CPC3000
Surface topography measurement	FRT 300 μm sensor
Analog readout device	NI I/O-Box 6211

**Table 3:** Micropump key parameters.

Actuator	Diaphragm Thickness	Diaphragm Diameter	Piezo Thickness	Piezo Diameter	Piezo Type	Pump Chamber Height
	40 μm	6.6 mm	150 μm	5.6 mm	PIC255	3 μm
Valves	Flap length	Flap width	Flap thickness	Valve seat length (cubic)	Seal lip width	
	800 μm	400 μm	15 μm	3 00 μm	6 μm	

**Table 4:** Differential Pressure Based (DPB) sensor key parameters.

Label	Pressure Range	Repeatability and Hysteresis	Nozzle Diameter	Diffuser Diameter	Max. Nozzle Flow	Max. Diffuser Flow
DPB65μ	5 psi = 34.5 kPa at 110 mV	±52 Pa or ±0.17 mV	65 μm	60 μm	270 μL/min	260 μL/min
DPB50μ	5 psi = 34.5 kPa at 110 mV	±52 Pa or ±0.17 mV	50 μm	45 μm	165 μL/min	158 μL/min

**Table 5:** Measurement methods overview.

Method	Measurement of
(a) Static calibration of flow sensors	Static reference flow vs. sensor voltage
(b) Dynamic calibration of flow sensors	Stroke volume vs. sensor voltage vs. average reference flow
